# Incidence and predictors of attrition rate after children started inpatient treatments for complicated severe acute malnutrition in North West Ethiopia

**DOI:** 10.1186/s41043-022-00332-8

**Published:** 2022-11-29

**Authors:** Fassikaw Kebede, Tsehay Kebede

**Affiliations:** 1 Department of Epidemiology and Biostatics, School of Public Health, College of Health Science, Woldia University, Woldia Town, Street, Technique, Kebele-05, P.O. Box: 400, Woldia, Ethiopia; 2 Department of Geography and Environmental Studies, Faculty of Social Science, Bahir Dar University, P.O. Box: 72, Bahir Dar, Amhara Ethiopia

**Keywords:** Attrition rate, Defaulter, Children, Cox regression, Severe acute malnutrition, Retrospective study, Ethiopia

## Abstract

**Background:**

Retaining children for inpatient treatment of complicated severe acute malnutrition (SAM) is a growing challenge until achieved the reference weight of a child. In Ethiopia, there is limited information regarding the time to be lost from the stabilizing centers after initiation of treatment. Thus, this study aimed to identify incidence and predictors of attrition for children suffering from SAM after started inpatient treatment in North West Ethiopia.

**Methods:**

A retrospective cohort study was conducted among under-five children admitted and started inpatient treatment for complicated SAM from 2015/2016 to 2020/2021. Data were entered using Epi-data version 4.2 and then exported to STATA (SE) version R-14 software for further analysis. The analysis was computed using Cox proportional hazard regression model after checking all proportional hazard assumptions. Covariates having < 0.2 of *P* values in the bi-variable analysis were candidates transferred to the multivariable Cox proportional hazard regression model. Finally, a statistical significance was declared at a *P* value of < 0.05.

**Result:**

Overall, 760 files of under-five children were analyzed with a mean (± SD) age of participants 27.8 (± 16.5) months. About 6944 child-days of treatment observation were recorded with the crude incidence of attrition rate of 9.7% (95% CI 7.9–12.6). The overall median time of attrition and half-life time *S*(*t*_1/2_) of survival rates was determined as 14 (IQR =  ± 7) days and 91.6% (95% CI 88.2–93.1), respectively. The attrition rate was significantly associated with cases living in rural residents (AHR = 6.03; 95% CI 2.2; 25.2), being re-admitted SAM cases (AHR = 2.99; 95% CI 1.62; 5.5), and caregivers did not have formal education (AHR = :5.6, 95% CI 2.7; 11.7) were all independent predictors for attrition from inpatient treatment.

**Conclusions:**

Nearly one in every ten severely acute malnourished under-five children defaulted at the end of treatment observation with a median time of 14 (IQR =  ± 7) days. Living in a rural residence, being re-admitted cases, caregivers who did not have a formal education were significantly associated with the attrition rate. Hence, it is crucial to detect and control the identified causes of defaulting from treatment observation promptly. Furthermore, serious counseling during admission and nutritional provision strategies are essential for virtuous treatment outcomes.

## Introduction

Severe acute malnutrition (SAM) is defined by the World Health Organization (WHO) as the presence of at least one of these three independent criteria: < − 3 Z-score weight-for-height (WHZ), a mid-upper arm circumference (MUAC) of < 115 mm, and bilateral pitting edema (known as ‘kwashiorkor’) [1]. SAM is a worldwide problem and one of the top deadly diseases for children less than 5 years of age and children with SAM have nine times more mortality rates than their peers do have [2]. It is responsible directly or indirectly for 60% of the 10.9 million deaths annually among under-five children, and two-thirds of these deaths occur during the first year of life [3–5]. When a child has SAM, he or she often suffers from lifelong repercussions throughout his or her life, passing on the dreadful legacy to future generations [6, 7].

Globally, in 2018, one in 12 of the estimated 52 million children under five had SAM and 2.9 million of these children were admitted for inpatient treatment [6–8]. However, attrition is the interruption of SAM inpatient treatment before reaching the target weight for at least two consecutive days and it is a composite term for defaulted and self-discharged cases [4, 9]. According to international Sphere standards, there are three main exit criteria for inpatient treatment of complicated SAM including cured, attrition, and death [6, 10]. However, previous study finding indicated that about 20% to 36% self-discharged after treatment initiated was responsible for incidence of 1.2% to 10.4% re-admission of cases [3, 4, 9, 11].

Accordingly, factors like number of family in a house, caregivers’ educational status, distance from health institutions, lack of transport access and fees were identified as primary causes for the interruptions of inpatient treatment [12, 13]. It may also be associated with healthcare providers’ negative attitudes toward long waiting times, drug side effects, and lack of social support [3, 4, 9]. Potentially*,* children who drop out of inpatient treatment before attaining the reference weight have a higher risk for re-admission (relapse) and mortality than those children discharged as cured [8, 14–18]. In Ethiopia, there is limited information regarding the predictors of attrition rate from stabilizing centers. This work aims to identify factors associated with the attrition rate after being admitted for inpatient treatment give insight for cause, and estimated time for attrition rate for children suffering from complicated SAM in Pawe General Hospital, North West Ethiopia.

## Methods

### Study areas and settings

This study was conducted at the Pediatric ward of Pawe referral hospital. This hospital is located in the Metekel Zone in North West of Benishangul Gumuz regions. This region is one of the eleven national regional states in Ethiopia with three administrative zones; Metekel, Kamshi, and Assosa zones. Pawe hospital is located, 364 km away from Assosa, the regional capital city, and 565 km from Addis Ababa the national capital city at Metekel zone within 34° 10′ N and 37° 40′ E and latitude 09° 17′ N and 12° 06′ N.

### Study design

Hospital-based retrospective cohort study was employed among 760 under-five children those were admitted for treatment of complicated SAM since January 1, 2015 to December 31, 2020.

### Source and study population

All under-five children with complicated SAM and admitted for inpatient treatment for at least 1 day of observation from January 1, 2015 to December 31, 2020 were included.

### Inclusion criteria

All under-five children admitted for complicated SAM and started inpatient treatment since 1 January 2015 to 31 December 2020 in Pawe referral hospital were included for this study as study subject. However, a child whose final treatment outcomes were not defined was excluded even if admitted and started the inpatient treatment in the wards.

### Sample size determination

The sample size was calculated based on the formula for double population proportions using open EPI-Info version 2.3.1. The formula considers the following parameters, levels of significant 5%, power of the study of 80%, and risk ratio of 1.35, and the outcome of the unexposed group of 27.1% was taken [7]. The total sample size to detect the factors lost to follow-up after starting SAM treatment care in the stabilizing center was 724 (including 10% with incomplete records and computed as final sample 760).However, from January 1st, 2015 to December 31st, 2020, only 760 inpatient admitted children were treated for complicated SAM in Pawe referral Hospital. There for since the final sample size is manageable in resource we included all file rather than applying sampling procedures.

### Outcome ascertainment

The dependent variable for this study was attrition (defaulting) as an event of interest before reached on the target weight. Censored; when a child was started the inpatient treatments for complicated SAM and declared cured at weight-for-height/length is ≥ − 2 Z scores, waiting without edema for at least 2 weeks/and a MUAC > 115 mm, and no edema for at least 2 weeks. The categorical variables like socio-demographic characteristics of children and caregivers, and clinical and medical descriptions of children were independent.

### Operational words

Complicated severe acute malnutrition (CSAM): it is defined by the World Health Organization (WHO) as the presence of at least one of these three independent criteria: < − 3 Z-score weight-for-height (WHZ), a mid-upper arm circumference (MUAC) of < 115 mm, and bilateral pitting edema (commonly known as ‘kwashiorkor and failed appetite test [3, 19]. Discharged/declared cured: this was defined as a child whose weight-for-height/length is ≥ − 2z scores without edema for at least 2 weeks or a MUAC > 115 mm and no edema for at least 2 weeks [3, 19].

Attrition (default/dropout/loss from follow-up); where a child who was not seen for at least two consecutive days after being admitted and started complicated SAM inpatient treatment with or without treatment progression [3, 19]. Relapse rate/re-admission rate; the proportion of children who re-enrolled after they recovered and were discharged.

### Data collection instruments and quality control

A standard and pretested data extraction tool was used to extract the required information from the case notes both for new and re-admitted cases. Before the actual data collection, the prepared checklist of variables was pretested in 28 case notes of HIV-infected children at Jawi Primary hospital. The 2-day training was given for the two diploma nurses data collectors and for a degree public health officer with the objective of study outcome and maintaining data confidentiality. An assigned supervisor strictly followed and oversaw the completeness of the collected data and feedback was given daily.

### Data processing and analysis

The data were entered into Epi-data version 4.2, and exported to STATA (SE)/14 for analysis. Before analysis, the data were cleaned, and simple frequency, cross-tabulation, and categorization of continuous variables were done. The WHO Anthro-Plus-version 1.04and ENA for Smart Software was used to generate the Z score (WAZ, HAZ, WHZ/BAZ) to define the nutritional status of seropositive children. Descriptive nonparametric survival analyses such as the life table and Kaplan–Meier survival curve were used to estimate the cumulative probability of developing SAM and the median time to develop SAM during successive follow-ups, respectively. The Kaplan–Meier plot compared the survival times for two or more group categories on the SAM graph to detect a difference in new or re-admission cases.

Assessing whether or not there is a real statistically significant difference between the two groups will be tested by using the log-rank test. Under the log-rank test, the null hypothesis (there is no difference between the survival times of the two groups) is tested against the alternative that the survival times are not the same among categories, and the stratum of covariates was considered statistically significant at the *P* value 0.05 in the log-rank test. Finally, we used *Cox proportional* hazards regression model with robust sandwich covariance matrix estimates to account for repeated measurements for each malnourished child. Before running multiple *Cox proportional regression*, the test of proportionality hazard assumption was checked using graphical methods (*log–log plot*) and statistical methods (*global goodness of fit test*, time-dependent). The final analysis was computed using Cox proportional hazard regression model after checking all the above assumptions. Covariates having < 0.2 *P* values in the bi-variable analysis were fitted to Cox proportional multivariable model. Finally, statistical significance was declared at a *P* value of < 0.05.

## Result

### Socio-demographic characteristics of SAM admitted children

Overall, 760 files of under-five children were analyzed. The majority 427 (56.18%) of the respondents were female in gender, and 556 (73.16%) of them were rural inhabitants. The overall mean (± SD) age of participant children was found to be 27.8 (± 16.5) months, with more than half of 458 (60.26%) of the cases ≤ 24 months. This study also showed that 244 (32.11%) of caregivers had no formal education; however, more than half of 465 (61.18%) mothers were on breastfeeding for their dyads at admission. The overall mean (± SD) weight and MUAC of participant cases at admission were 11.3 (± 23.6) kg and 10.7 (± 1.4) cm, respectively (Table 1).

**Table 1:** Baseline socio-demographic and clinical characteristics of under-five children during SAM inpatient treatment in Pawe Hospital

Variables	Categories	Frequency	Percent
Age	6–24 months	458	60.26
	25–48 months	219	28.82
	48–60 months	83	10.92
Gender	Male	333	43.82
	Female	427	56.18
Resident	Rural	556	73.16
	Urban	204	26.84
Maternal education status	Formally educated	516	67.89
	Had no formal education	244	32.11
Anemia	≥ 10 mg/dl	511	67.24
	< 10 mg/dl	249	32.76
Nasogastric intubation	Yes	318	41.84
	No	442	58.16
Vomiting	Yes	539	70.92
	No	221	29.08
Diarrheas	Yes	409	53.82
	No	351	46.18
Types of malnutrition	Marasmus	431	56.71
	Marasmus–Kwashiorkor	230	30.26
	Kwashiorkor	99	13.03
Blood transfusion	Yes	168	22.11
	No	592	77.89
Breastfeeding	Yes	465	61.18
	No	295	38.82
Edema grading	No	425	55.92
	Grade+	277	36.45
	Grade++	40	5.26
	Grade+++	18	2.37
Vitamin A supplementation	Yes	584	76.84
	No	176	23.16
Folic acids supplementation	Yes	525	69.08
	No	235	30.92
MUAC	≤ 115 mm	131	17.24
	> 115 mm	629	82.76
Pneumonia	Yes	452	59.47
	No	308	40.53
TB	Yes	45	5.92
	No	715	94.08
Number of families	≤ 2	243	31.97
	3–4	419	55.13
	≥ 5	98	12.89
Admission types	New	685	90.13
	Re-admission	75	9.8
Deworming	Yes	331	43.55
	No	429	56.45
Amoxicillin	Given	624	82.11
	Not given	136	17.89
Vaccination status	Completed	572	75.26
	Not completed	188	24.74
HIV infection	Positive	42	5.53
	Negative	718	94.47
Skin infection	Yes	290	38.16
	No	470	61.84
Zink supplementation	Yes	349	45.9
	No	411	54.08
IV fluid given/transfusion	Yes	190	25.00
	No	570	75.00
IV antibiotics	Missed/not given	567	74.61
	Given	193	25.39
Ampicillin and/or Gentamicin	Given	604	79.47
	Not given	156	20.53
F-75 formula milk	Completed	734	96.5
	Not completed	26	3.42
F-100 formula milk	Completed	715	94.1
	Not completed	45	5.9

### Baseline comorbidities and medications

Furthermore, nearly three in five of 452 (59.47%) of admitted cases a complication of severe pneumonia, whereas the largest proportions of 718 (94.47%) under-five children were negative for HIV tests. Likewise, more than half 431 (56.7%) of participant children were admitted through marasmus, while the remaining 230 (30.26%) and 99 (13.03%) cases were treated as mixed (marasmic-kuash), and edematous (kwashiorkor), respectively. The study also showed that 244 (32.11%) caregivers of children had no formal education, of which 66 (9.2%) children defaulted from inpatient treatment (Table 1).

### Incidence of attrition rate

The 5-year retrospective cohort study of 760 cases yielded 6944 days of risk observation. During the follow-up period, 629 (82.8%) children were cured, while 39 (5.4%) died, 71 (9.34%) defaulted, and 22 (2.83%) were medically transferred to other health institutions. The overall crude incidence of attrition rate was found to be 9.7 per 100 child weeks (95% CI 7.9–12.6).

### Kaplan–Meier hazard curve and survival difference

**Fig. 1:**
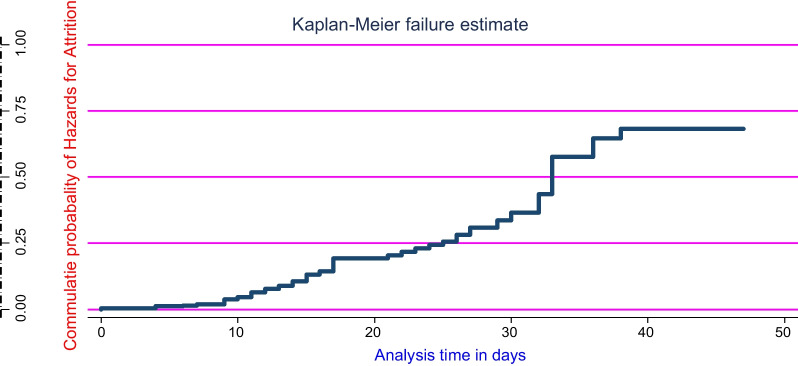
Hazard estimate of attrition rate for under-five children after started inpatient treatment for complicated SAM Pawe referral Hospital

During inpatient treatment, cases were from rural, with no formal education of caregivers, and having >= 5 number of family in a house were  early experienced an interruption of the  treatment, tested on the long rank test at *P* < 0.05. Accordingly, the overall hazard curve of the follow-up was stepwise, upwardly, and crossed the survival curve when time increased exactly at a half-life time of 0.5 (Fig. 1).

**Fig. 2:**
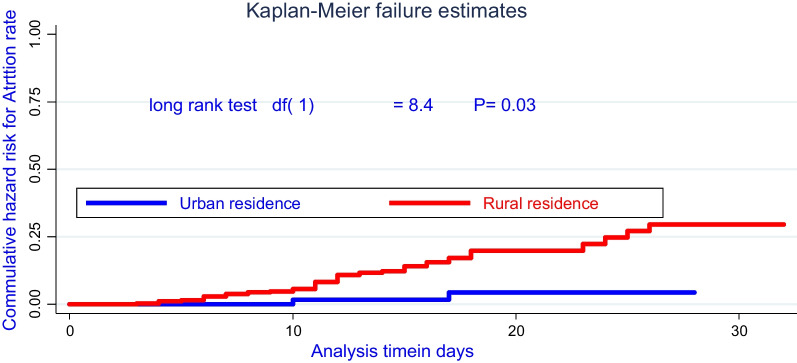
Comparing hazard of attrition rate by permanent residence for under-five children treated on SAM suffering with the complication at Pawe referral Hospital

**Fig. 3:**
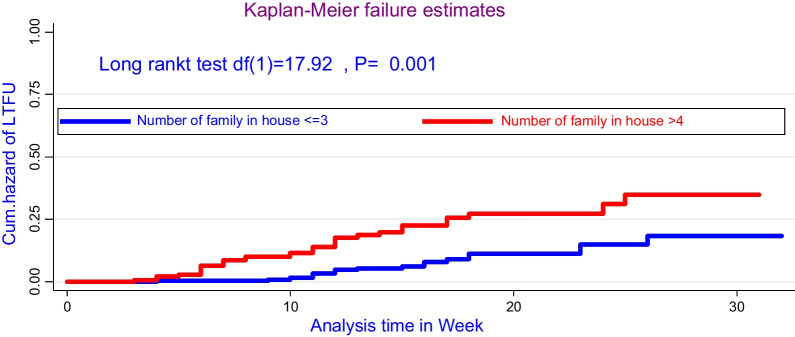
Compares the hazard of attrition rate by family size for under-five children treated on SAM suffering from the complication at Pawe referral Hospital

**Fig. 4:**
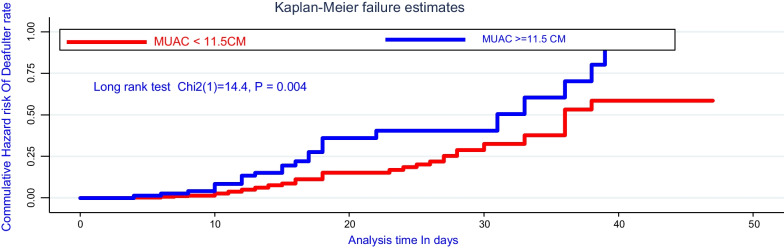
Comparing hazard of attrition rate by baseline MUAC of under-five children treated on SAM suffering from the complication at Pawe referral Hospital

Moreover, there was a significant survival difference in attrition rate for admitted cases of rural and urban, where about being rural children was early defaulted from treatment center as compared with urban cases, evidenced by the log-rank test (*χ*^2^; *df*(1) = 8.40, *P* = 0.03). In addition to this, there was a significant survival difference of attrition among cases living with > 5 family sizes in a house was early interrupted from inpatient treatment as compared with ≤ 2 family and evidence on the log-rank test (*χ*^2^: (*df* = (2) = 17.9, *P* = 0.001) as shown in Figs. 1, 2, 3, and 4.

### The log-rank estimate of defaulter rate and life tables of cases

We have retrospectively followed children for a minimum of 3 days and a maximum of 48 days of observation with a median (± IQR) follow-up time of 14 (IQR ± 7) days. Of the total 71 (9.34%) defaulted cases, the majority 45 (66.8%) of those were attrite after 14 days of treatment initiation.

The survival rate in the report ate half-life time was 91.66% (95%CI 88.2–93.1). The overall incidence density of attrition rate (IDR) was reported as 5.8 (95% CI 4.4–7.56) with a mean (± SD) time of inpatient hospital stay was 15.8 (± 7.37) days (Table 2).

**Table 2:** Life table showing the cumulative survival probability for time to and predictors of under-five children attritions from the treatment center

Days	No. of children at initial	Defaulted cases on each interval days	Cumulative defaulter	Remaining at-risk children	Proportions of attrition on each interval (%)	Survival rate	95% CI
1–7	760 (100)	15 (21.2)	15 (21.2)	745 (98.1)	1.99	0.9798	0.96; 0.98
8–14	745 (98.02)	32 (45.1)	47 (66.2)	713 (93.8)	4.2	0.9166	0.88; 0.93
15–21	713 (93.8)	16 (23.5)	63 (88.7)	696 (91.5)	2.23	0.8356	0.78;0.87
22–28	696 (91.5)	7 (9.93)	70 (98.6)	689 (90.6)	0.91	0.7293	0.63; 0.80
≥ 29	689 (90.6)	1 (0.13)	73 (100)	688 (90.2)	0.12	0.6772	0.53; 0.78
Total	688 (90.6)	71 (100)	71 (100)	688 (90.2)	9.42	0.6772	0.53; 0.78

### Bi-variable and multivariable Cox regression analysis

During bi-variable *Cox regression* analysis, variables were checked whether they were associated with the incidence of attrition rate at *P* value < 0.2 for a candidate selection of multivariable *Cox regression*. After adjusting certain confounding, ninety variables were fitted to build the final model. On the other hand, permanent residents, caregivers’ level of education, and admission type were significantly associated with attrition (lost from follow-up) incidents at 5%levels of significance. Accordingly, the hazards of attrition (defaulting) from SAM inpatient treatment for rural under-five cases were 6.08 times increased as compared with the urban group (AHR = 6.1: 95% CI 2.2; 26.2, *P* = 0.013). Likewise, the attrition (defaulting) rate was significantly associated with re-admitted SAM cases. Being re-admitted SAM cases nearly three times increased the risk of defaulting after initiating SAM inpatient treatment (AHR = 2.99, 95% CI 1.62; 5.49, *P* = 0.001). Moreover, the attrition rate was significantly higher for caregivers who had no formal education, whereas caregivers of the admitted cases were no formal education for nearly six-time increase the risk of attrite or loss from follow-up (AHR = :5.6, 95% CI 2.76; 11.7, *P* = 0.001) as compared with a counter group (Table 3).

**Table 3:** Bi-variable and multivariable Cox regression to determine predictors of attrition rate from SAM inpatient treatment center

Variables	Categories	Frequency	Survival status of SAM cases		CHR	AHR	*P* value
			Attrition (71)	Censored			
Age	≥ 24 months	458 (60.26)	42 (5.52)	416 (54.7)	1	1	
	25–48 months	219 (28.82)	22 (2.89)	197 (25.9)	1.2 (.68;2.2)	1.19 (0.6;2.2)	0.27
	48–60 months	83 (10.92)	7 (0.92)	76 (10.1)	2.11 (.86;1.9)	1.8 (0.7:1.5)	0.11
Sex	Male	330 (43.82)	47 (6.18)	283 (37.2)	2.3 (1.3:4.1)	1.56 (.85;2.78)	0.14
	Female	430 (56.18)	24 (3.15)	406 (53.4)	1	1	
Resident	Rural	570 (74.16)	6 (0.78)	184 (24.2)	6.3 (1.5;26.1)	6.3 (1.6;26.4)	0.012*
	Urban	190 (27.84)	65 (8.55)	505 (66.4)	1	1	
Anemia	≥ 10 mg/dl	511 (66.24)	20 (2.6)	482 (63.4)	1	1	
	< 10 mg/dl	258 (33.76)	51 (6.7)	207 (27.2)	5.1 (2.8;9.3)	1.7 (0.9:2.1)	0.16
Diarrheas	Yes	409 (53.82)	33 (4.4)	376 (49.4)	1.7 (0.77;2.9)	1.2 (0.8;2.4)	0.22
	No	351 (46.18)	38 (5.0)	313 (41.1)	1	1	
Vitamin A	Given	584 (76.84)	56 (7.36)	528 (69.4)	1.3 (.67;2.5)	1.5 (.7;2.6)	0.12
	Not given	176 (23.16)	15 (1.9)	161 (21.1)	1	1	
Folic acids	Given	525 (69.08)	50 (6.57)	475 (62.5)	1.3 (0.7;2.3)	1.3 (0.6;2.4)	0.34
	Not given	235 (30.92)	21 (2.7)	214 (28.15)	1		
Pneumonia	Yes	452 (59.47)	61 (8.1)	397 (52.2)	2.9 (2.4;10.3)	1.2 (0.8;3.2)	0.08
	No	308 (40.53)	10 (1.3)	292 (38.4)	1	1	
TB	Present	45 (5.92)	6 (0.78)	39 (5.13)	1.1 (0.26;4.5)	1.1 (0.3;1.2)	0.3
	Absent	715 (94.08)	65 (8.5)	650 (85.5)	1	1	
Family size	≤ 4	487 (64.97)	25 (3.2)	462 (60.78)	1	1	
	> 5	273 (35.1%)	46 (6.1)	227 (29.8)	3.1 (1.7;5.4)	0.9 (.7;1.4)	0.12
Deworming	Yes	331 (43.55)	36 (4.7)	295 (38.8)	1		
	No	429 (56.45)	35 (4.6)	394 (51.8)	1.2 (0.68;2.0)	1.1 (0.7;2)	0.11
Amoxicillin	Given	624 (82.11)	62 (8.2)	662 (87.1)	1	1	
	Not given	136 (17.89)	9 (1.1)	127 (16.7)	0.9 (.43;1.95)	0.9 (.43;1.9)	0.11
Vaccine status	Completed	572 (75.26)	54 (7.1)	518 (68.1)	1	1	
	Not completed	188 (24.74)	17 (2.2)	171 (22.5)	1.3 (.69;2.64)	1.1 (.5;2.2)	0.41
HIV infection	Positive	42 (5.53)	5 (0.6)	37 (4.86)	1.1 (.45;2.9)	1.1 (.3;2.1)	0.22
	Negative	718 (94.47)	66 (8.6)	652 (85.78)	1	1	
Skin infection	Yes	290 (38.16)	36 (4.7)	254 (33.4)	1.4 (.80;2.4)	1.2 (.6;2.1)	0.15
	No	470 (61.84)	35 (4.6)	435 (57.2)	1	1	
Zink supplement	Yes	349 (45.9)	33 (4.3)	373 (49.1)	1	1	
	No	411 (54.08)	38 (5.0)	316 (41.5)	1.2 (.64;1.9)	0.8 (.6;1.9)	0.31
Maternal education	Formally educated	516 (67.89)	16 (2.2)	500 (65.7)	1	1	
	Had No formal education	244 (32.2)	55 (7.2)	189 (24.8)	9.6 (4.8;19.2)	5.3 (2.6:10.9)	0.01*
Admission types	New	685 (90.13)	42 (5.5)	643 (84.6)	1	1	
	Re-admission	75 (9.8)	29 (3.8)	46 (6.1)	4.9 (2.7;8.4)	2.8 (1.5;5.3)	0.01*

*indicated significant variables for an risk factors for Attrition rate

## Discussion

The final report of this study revealed that the overall crude incidence of attrition rate was found to be 9.34% (95% CI 7.5%: 11.6%). This is inconsistent and lower than the previous description of 16.5% in North West Ethiopia [20], and 24.1% in Sokoto—Nigeria [2]. However, this report is higher than an earlier narration of 2.2% in the Hadiya zone—Southern Ethiopia [3, 4]. The possible reason for the variation can be due to the differences in the study period, settings, sample size, and counseling barriers of healthcare providers during inpatient treatment. Moreover, the qualities of clinical healthcare service specifically the cost of the accessorial drug at baseline admission intended to caregivers self-discharged. On the other hand, early healthcare-seeking behavior and commitment of family for their children influence the length of hospital stays. What is more with report the mean (± SD) time of hospital stay with treatment observation in our study was estimated as 16.3 (± 7.6) days, which is lower than the national protocol of maximum length of inpatient stay to be 8 weeks for complicated cases [21]. This may be due to contributions of underlying-morbidities, and quality of healthcare providers’ on different health institutions. Regarding predictors for attrition rate, variables like residence, admission type, and maternal education status were significantly associated with attrition from stabilizing centers.

Consistent with previous findings in south Gondar [5, 22], the hazards of attrition rate from stabilizing center for rural cases of under-five children were 6.08 (95% CI 2.2; 25.2, *P* = 0.013) time increased as compared with their peers of urban class. The possible elucidation that may be topographically our study setting of Metekel Zone is a home land for refugee populations from Sudan and South-Sudan, in addition to be center for ethnic conflict zone [7, 23, 24], this makes the reginal population to be food insecured and significantly associated with excess re-admission of cases. Specifically, Gumuz population was reluctant to be stabled until their dyads declared cured due to continual cost of accessory drugs [18]. This makes them highly experienced self-discharged with or without treatment progression after started.

Moreover, the risk of attrition rate for re-admitted under-five children was nearly three 2.99 (AHR = 2.99, 95% CI 1.62; 5.49, *P* = 0.001) time increased as compared with new admitted SAM cases. This is consistent with previously reported in Pawe Hospital [6, 7, 18]. This might be due to the number of families in a house can cause interruptions from treatment observation. This may be a cause fear for care on transportation and feeding of schoolchildren at home [25, 26]. Consistent to this narration, our report confirmed that majority 49 (69.1%) of interrupted cases were lived with ≥ 5 number of families in a house.

Furthermore, the risk of attrition rate for caregivers of admitted children who had no formal education was nearly six (AHR = :5.6, 95% CI 2.76; 11.7, *P* = 0.001) times increased as compared with the counter group. This is consistent with previously reported in Gondar hospital [3, 27], Eastern Ethiopia [21], in Minia Hospital [28]. The possible reason for similarity might be educated mothers have an awareness regarding their child’s health, and they might be active unless SAM treatment is completed and before children obtained the targeted weight will not interrupt the ongoing treatment [15, 29]. Accordingly, in a bid to improve treatment outcomes for children with SAM and minimize attrition of caregivers, the WHO developed a ten-step guideline for effective management [29, 30], which is widely accepted for minimizing defaulters, there for endorsement of rules, and implementation strategies should be started earlier in the hospital.

## Limitations of the study

*First the retrospective nature of data collection missed significant variables like housel hold resource and food security status and might bias interpretations of the result. Second, we could get the defaulter and ask them about the reason for why coming up with the decision for lost follow-up, and we did not record the socioeconomic asset of caregivers at current levels. There this all may distort the result. Therefore, the interpretation and application of our findings for clinical decisions and policy should take into account these limitations in addition to lack of qualitative approaches for the same predictors.*

## Conclusion and recommendation

Nearly one in every ten severely acute malnourished (SAM) under-five children defaulted with a median time of 14 (IQR =  ± 7) days. Living in a rural residence, being re-admitted, caregivers who did not have a formal education were significantly associated with the attrition rate. Hence, it is crucial to detect and control the identified causes of defaulting from follow-up promptly. In addition, serious counseling for inpatient care and strengthened nutritional provision strategies are essential for virtuous treatment outcomes. There is a need to enhance community level interventions, targeted toward awareness dietary fortification and home-based therapy of malnutrition with RTUF to minimize high rate of lost follow-up (Attrition rate) after admission.

## Data Availability

The datasets employed in the current study are available with the corresponding author upon reasonable request via email.

## Abbreviations

AHR: Adjusted hazard ratio
CHR: Crude hazard ratio
CI: Confidence interval
FMOH: Ethiopian Federal Ministry of Health
MUAC: Mid-upper arm circumference
SAM: Severe acute malnutrition
WFH: Weight-for-height
SD: Standard deviation
